# Ginkgolic acid, a sumoylation inhibitor, promotes adipocyte commitment but suppresses adipocyte terminal differentiation of mouse bone marrow stromal cells

**DOI:** 10.1038/s41598-018-20244-0

**Published:** 2018-02-07

**Authors:** Huadie Liu, Jianshuang Li, Di Lu, Jie Li, Minmin Liu, Yuanzheng He, Bart O. Williams, Jiada Li, Tao Yang

**Affiliations:** 1 State Key Laboratory of Medical Genetics and School of Life Sciences, Central South University, Changsha, Hunan 410078 P. R. China; 2 Program of Skeletal Disease and Tumor Metastasis, Center for Cancer and Cell Biology, Van Andel Research Institute, Grand Rapids, MI 49503 USA; 3 Center for Epigenetics, Van Andel Research Institute, Grand Rapids, MI 49503 USA; 4 The Innovation and Integration Program, Center for Cancer and Cell Biology, Van Andel Research Institute, Grand Rapids, MI 49503 USA

## Abstract

Sumoylation is a post-translational modification process having an important influence in mesenchymal stem cell (MSC) differentiation. Thus, sumoylation-modulating chemicals might be used to control MSC differentiation for skeletal tissue engineering. In this work, we studied how the differentiation of mouse bone marrow stromal cells (mBMSCs) is affected by ginkgolic acid (GA), a potent sumoylation inhibitor also reported to inhibit histone acetylation transferase (HAT). Our results show that GA promoted the differentiation of mBMSCs into adipocytes when cultured in osteogenic medium. Moreover, mBMSCs pre-treated with GA showed enhanced pre-adipogenic gene expression and were more efficiently differentiated into adipocytes when subsequently cultured in the adipogenic medium. However, when GA was added at a later stage of adipogenesis, adipocyte maturation was markedly inhibited, with a dramatic down-regulation of multiple lipogenesis genes. Moreover, we found that the effects of garcinol, a HAT inhibitor, differed from those of GA in regulating adipocyte commitment and adipocyte maturation of mBMSCs, implying that the GA function in adipogenesis is likely through its activity as a sumoylation inhibitor, not as a HAT inhibitor. Overall, our studies revealed an unprecedented role of GA in MSC differentiation and provide new mechanistic insights into the use of GA in clinical applications.

## Introduction

Sumoylation is a post-translational modification in which small ubiquitin modifiers (SUMOs) are conjugated to protein targets by the E1, E2, and E3 sumoylation enzymes. The SUMO-specific protease family (SENP) desumoylases can remove SUMO modifications from proteins^1,2^. Sumoylation and desumoylation are involved in a variety of cellular processes such as nuclear-to-cytosolic translocation, transcriptional regulation, apoptosis, protein stability, response to stress, and stem cell/progenitor maintenance, pluripotency, and differentiation^2–6^. Mesenchymal stem cells (MSCs) are able to renew themselves and give rise to bone, cartilage, fat, etc., thus holding a promise of cell therapy and tissue engineering^7^.

Our current understanding of sumoylation in osteogenesis remains limited, and the available results are somewhat inconsistent. UBC9 (ubiquitin conjugating enzyme 9), the only known E2 sumoylation enzyme in cells, negatively regulates osteoblastic differentiation induced by BMP (bone morphogenetic protein), partially via sumoylation of SMAD4^8^. Recently, it was reported that the desumoylase SENP3 is associated with MLL1/MLL2 complexes and desumoylates RBBP5, thus activating a subset of HOX genes that regulate osteoblast differentiation^9^. In contrast, knocking down an isoform of PIAS2, which is an important E3 SUMO ligase, can markedly reduce the expression of osterix (OSX, a key osteogenic transcription factor), thus suppressing osteoblastic differentiation and matrix mineralization^10^. Also, *Ubc9* silencing can inhibit BMP signaling in a dose-dependent manner by decreasing SMAD4 and p-SMAD1 levels, leading to a reduction of RUNX2 expression^11^.

The relationship between sumoylation and adipogenesis also remains controversial. Some evidence suggests that sumoylation promotes adipogenesis. For example, i) *Sumo1*-null mice on a high-fat diet gained less weight, had fewer and smaller adipocytes, and had decreased PPARγ target gene expression^12^; ii) a reduced level of UBC9 in 3T3-L1 adipocytes caused a significant delay in PPARγ and C/EBPα expression; and iii) UBC9 has been found to regulate glucose transporter 4 (GLUT4) turnover in adipocytes^13^. On the other hand, some studies suggest that sumoylation pathways suppress adipogenesis. As examples, the expression of the desumoylase SENP2 is markedly increased upon adipogenesis induction, and its knockdown causes a reduction of C/EBPβ protein without affecting the mRNA level^14^. SENP1 enhances adipogenesis through SHARP-1 desumoylation, thus enhancing PPARγ expression and adipocyte differentiation^15^. In addition, several transcription factors regulating adipogenesis are sumoylation targets: for instance, the transcriptional activity of PPARγ, C/EBPα, and C/EBPβ can be negatively regulated by sumoylation^16,17^, and the sumoylation of KLF5 regulates the transcriptional programs of lipid metabolism^18^. Overall, these varied actions of the sumoylation process in osteogenesis and adipogenesis reflect the diversity of sumoylation targets and the complexity of MSC differentiation.

Recently, altered sumoylation has been implicated in cancer development and in aging (reviewed in^19,20^), suggesting that sumoylation-modulating chemicals might be of value for disease treatment. In fact, a few sumoylation inhibitors have been used for this purpose. Ginkgolic acid, a natural component extracted from *Ginkgo biloba* leaves that directly binds to and inhibits the sumoylation E1 enzyme (SAE1/SAE2)^21^, has shown a promising effect in suppressing cancer cell growth and migration^22–25^. Because sumoylation plays an important role in regulating stem cell maintenance and differentiation, we are motivated to study the function of sumoylation-modulating chemicals in MSC differentiation. Here, we report the role of ginkgolic acid in the osteogenesis and adipogenesis of MSCs.

## Results

### GA blocked mBMSC osteogenic differentiation

**Figure 1:**
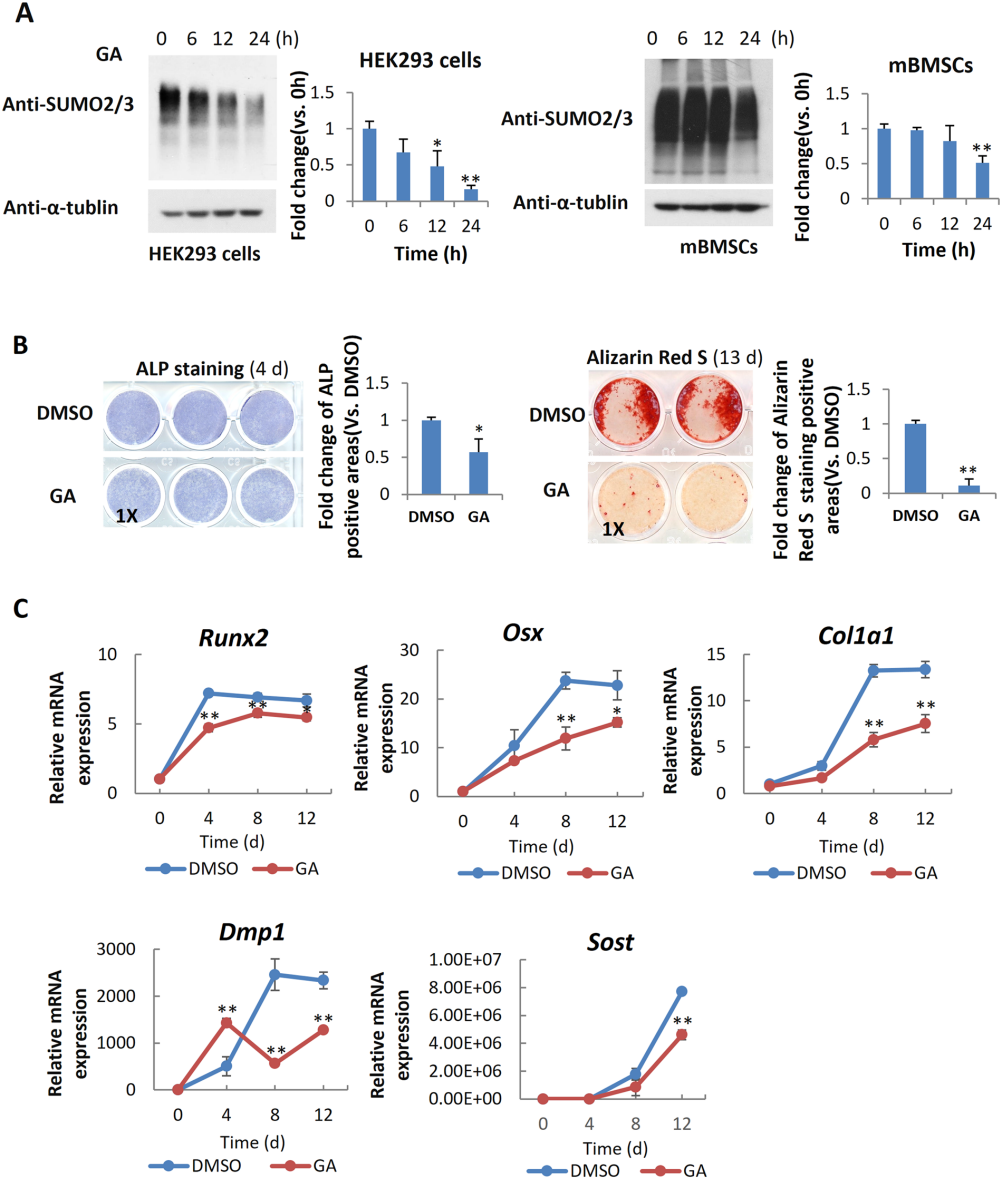
GA inhibited mBMSC osteogenic differentiation. (**A**) GA treatment (50 μM) led to a decrease of total sumoylated proteins in HEK293 cells and mBMSCs (*p < 0.05, **p < 0.01; n = 3). (**B**) GA treatment inhibited early and late osteogenic differentiation in mBMSC cultures as indicated by ALP staining and Alizarin Red S staining, respectively, and quantified on the right. (*p < 0.05, **p < 0.01; n = 3). (**C**) Treatment of mBMSCs in osteogenic cultures with GA (50 μM) caused a decrease in osteoblast and osteocyte differentiation markers. (*p < 0.05, **p < 0.01; n = 3).

To explore the gross effect of the sumoylation pathway in osteogenic differentiation, we chose primary mouse bone marrow stromal cells (mBMSCs) as an experimental model, because they are readily differentiated *ex vivo* into osteoblasts and adipocytes^26^. Ginkgolic acid (GA) was chosen to suppress the sumoylation pathway because it was reported as a potent inhibitor of the sumoylation E1 enzyme^21^. We confirmed the sumoylation-inhibiting activity of GA in both the HEK293 cells and mBMSCs (Fig. 1A).

We found that mBMSCs incubated in osteogenic differentiation medium with 50 µM GA showed a distinct decrease in both alkaline phosphatase (ALP) activity at day 4 and Alizarin Red S staining at day 13 (Fig. 1B). We also found that the expression of osteoblast markers *Runx2*, *Osx*, and *Col1a1* and the osteocyte markers *Dmp1* and *Sost* were all significantly decreased (Fig. 1C). These data suggested that GA blocked both the early and late osteogenic differentiation of mBMSCs.

### GA enhanced mBMSC adipogenesis under osteogenic induction

**Figure 2:**
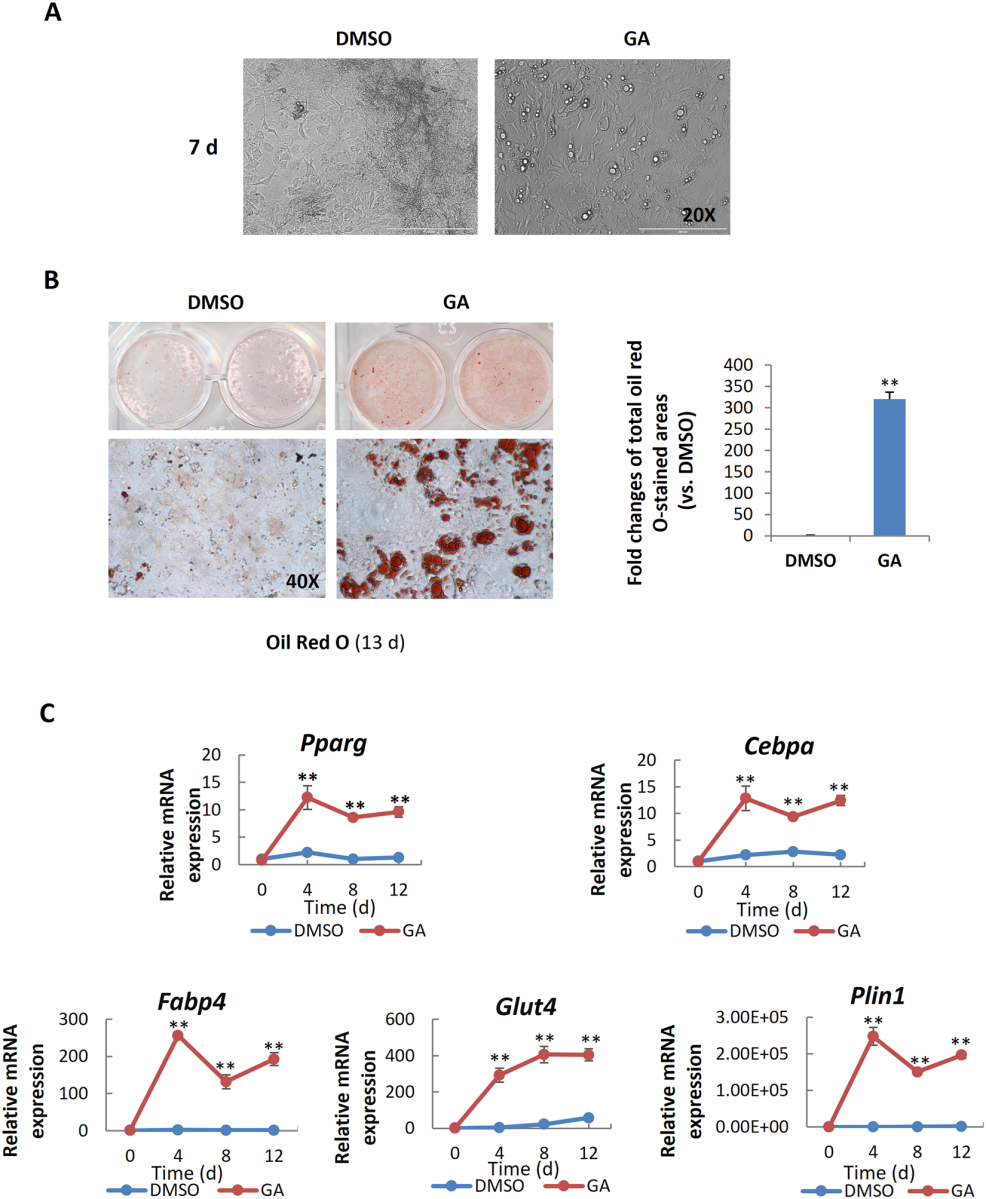
GA enhanced the adipogenesis of mBMSCs in osteogenic culture medium. (**A**,**B**) Phase-contrast microscopy (**A**) and oil red O staining (**B**) showed GA-induced lipid droplet formation in the mBMSC cultures at day 7 and 13 respectively in osteogenic medium; the oil red O–stained areas are quantified on the right (**p < 0.01; n = 3). (**C**) GA treatment increased the expression of key adipogenic markers (**p < 0.01; n = 3).

To our surprise, in osteogenic cultures we observed that many GA-treated mBMSCs contained bright, round vesicles reminiscent of lipid droplets (Fig. 2A); this was confirmed by oil red O staining (Fig. 2B). Moreover, the GA-treated mBMSCs cultured in osteogenic medium showed a markedly elevated expression of adipogenic regulators and adipocyte markers, including *Pparg*, *Cebpα*, *Fabp4*, *Glut4*, and *Plin1* (Fig. 2C), suggesting that GA robustly promoted mBMSCs adipogenesis even under osteogenic induction.

### Dexamethasone was required for GA-induced mBMSC adipogenesis

**Figure 3:**
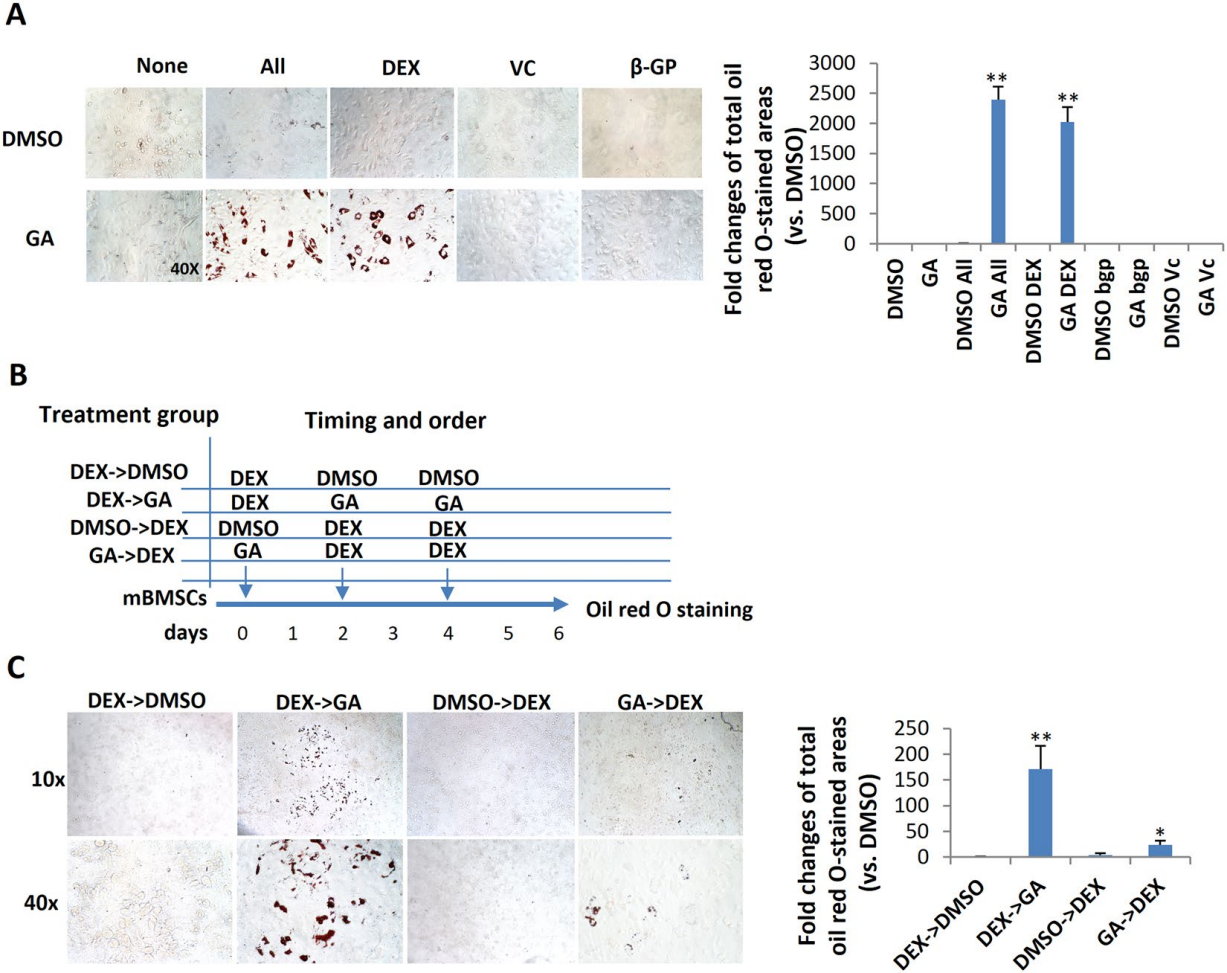
Dexamethasone potentiated GA-induced mBMSC adipogenesis. (**A**) Oil red O staining of mBMSCs cultured in GA (50 μM) with the complete osteogenic medium or individual components. The oil red O–stained areas are quantified on the right (**p < 0.01; n = 3). (**B**) The experimental scheme for DEX and GA treatment. (**C**) mBMSCs treated with DEX followed by GA (50 μM) showed enhanced adipogenesis. The oil red O staining of mBMSC cultures at day 6; the positively stained areas are quantified on the right (*p < 0.05, **p < 0.01; n = 3).

The above data suggest that GA may be able to switch the fate of mBMSCs from osteogenesis to adipogenesis, but it was unclear whether GA alone was sufficient or component(s) of the osteogenic medium were also required. To dissect this, we first treated mBMSCs with GA alone in normal culture medium for 6 d and found no adipogenesis (data not shown), suggesting that one or more component(s) in the osteogenic medium was required. Next, we treated cultured mBMSCs with GA plus each component of the osteogenic medium, including ascorbic acid (VC), β-glycerophosphate (β-GP), and dexamethasone (DEX). Our data showed that only DEX synergized with GA in adipogenic induction (Fig. 3A).

To understand the action sequence of GA and DEX in adipogenic fate determination, we first treated mBMSCs with DEX or GA for 48 h, then switched to the other compound for the following 4 days. DMSO was used as a control, and the treatment scheme is shown in Fig. 3B. We found that only the DEX-to-GA treatment produced large number of oil red O–positive cells; GA-to-DEX treatment produced few. The DEX-to-DMSO and DMSO-to-DEX treatments produced no oil red O–positive cells (Fig. 3C), suggesting that DEX is necessary for potentiating mBMSCs to a GA-induced adipogenic fate.

### Transcriptomic changes in mBMSCs co-treated with DEX and GA

**Figure 4:**
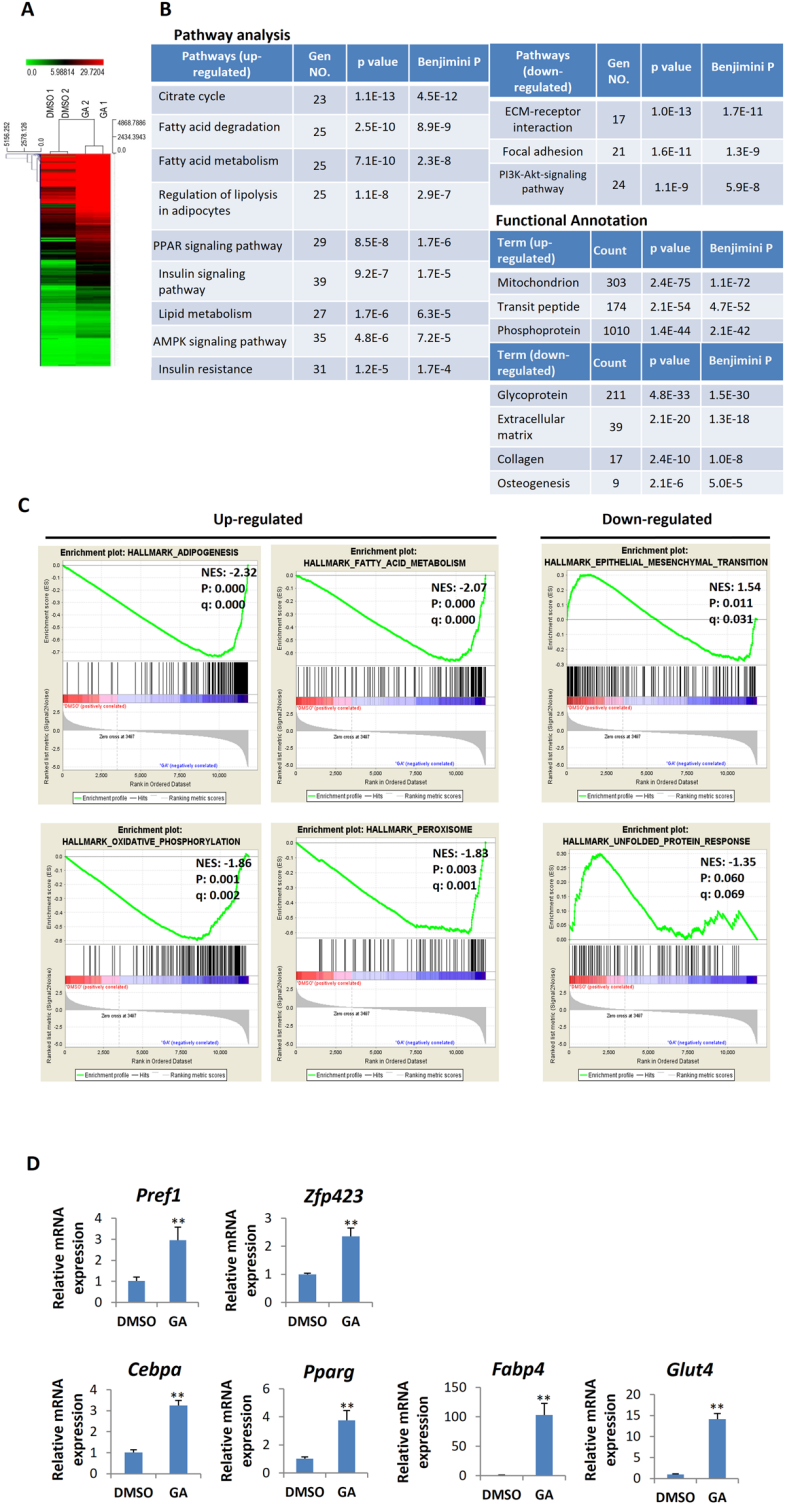
Transcriptomic changes in the mBMSCs caused by DEX-plus-GA treatment. (**A**) Heat map of differential gene expression in mBMSCs treated with GA (50 μM) plus DEX vs. DMSO plus DEX for 3 d (n = 2). (**B**) The most up-regulated and down-regulated pathways in the GA-plus-DEX treatment group revealed by DAVID GO analyses. Genes that showed significant difference between GA and DMSO treatments (p < 0.05) and had a log_2_ fold change > 0.5 or < –0.5 were chosen for analysis. (**C**) GSEA analyses of up-regulated and down-regulated gene sets (NES: normalized enrichment score; p: nominal p-value; q: false discovery rate q-value). (**D**) Altered expression of adipogenic genes in the mBMSCs treated with GA (50 μM) plus DEX vs. DMSO plus DEX for 4 d (**p < 0.01; n = 4).

To investigate how GA influences the fate of mBMSCs, we compared the transcriptomic profiles of mBMSCs under DEX-plus-DMSO vs. DEX-plus-GA treatment (3 d) by RNA sequencing (RNA-seq) (Fig. 4A). By pathway analysis using DAVID (Database for Annotation, Visualization and Integrated Discovery), we found that the citrate cycle, lipolysis and fatty acid degradation/metabolism, insulin resistance, and PPAR signaling were the most significantly enhanced, while ECM receptor interaction, focal adhesion, and the PI3K/Akt pathway were decreased. In addition, the expression of mitochondrial genes was markedly elevated, but the expression of osteogenesis, ECM, and collagen genes was decreased (Fig. 4B)^27^. GSEA (Gene Set Enrichment Analysis) also supported the finding that both the adipocyte differentiation and lipid metabolism pathways were enhanced (Fig. 4C)^28^.

From the list of up-regulated genes, we noticed that genes for a set of important adipogenic transcription factors, including a crucial transcription factor for adipocyte commitment *(Zfp423*) and pivotal transcription factors for adipocyte maturation (*Pparg*, *Cebpα*, and *Srebf1*), were dramatically increased. Lipogenesis-related genes such as *Fasn*, *Acaca*, *Aacacb*, *Dgat1*, and *Dgat2* were also markedly increased, and many key components of mitochondrial functions, including *Sdha*, *Cycs*, *Sod2*, and *Ucp2*, were increased by 2- to 3-fold. Consistent with the RNAseq data, mBMSCs cultured in normal medium containing both DEX and GA led to a significant increase in the expression of genes for markers of pre-adipocytes (*Zfp423* and *Pref-1*), for markers of committed adipocytes (*Pparg* and *Cebpα*), and for markers of mature adipocytes (*Fabp4* and *Glut4*), relative to cultures treated with DEX and DMSO (control) (Fig. 4D). These data overall echo our findings that GA promoted adipogenesis but blocked osteogenesis.

### GA enhanced mBMSC adipocyte commitment

**Figure 5:**
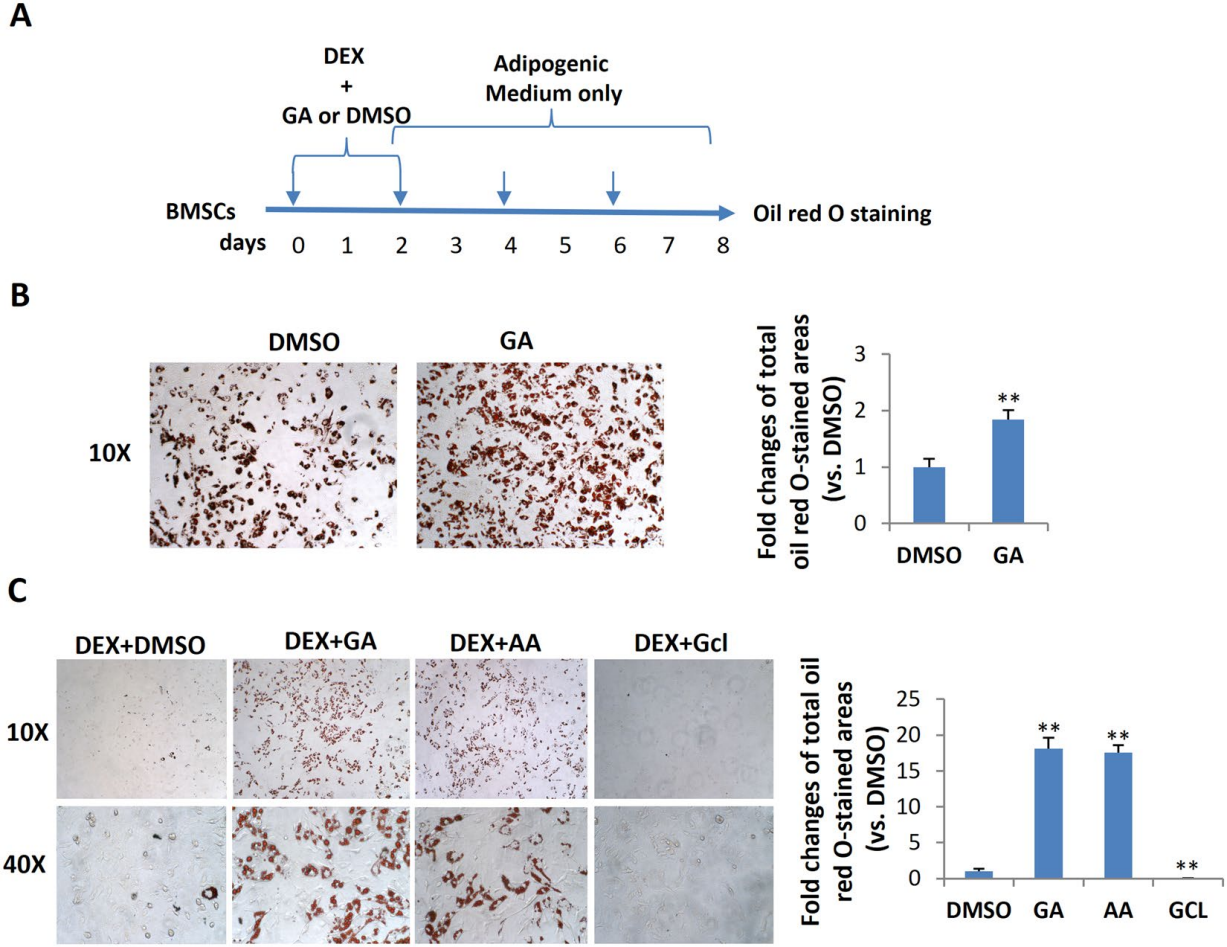
GA enhanced mBMSC adipocyte commitment. (**A**) The treatment scheme indicating the reagents, timing, and order. (**B**) Oil red O staining of the mBMSC cultures at day 8. The oil red O–stained areas are quantified on the right (**p < 0.01; n = 3). (**C**) mBMSCs co-treated with DEX plus GA (50 µM) or DEX plus AA (50 µM) (but not DEX plus Gcl (7 µM)) potentiated adipogenesis as indicated by oil red O staining. The oil red O–stained areas are quantified on the right (**p < 0.01; n = 3).

The adipogenic differentiation of MSCs has two phases. The first phase is adipogenic commitment, i.e., the differentiation of pre-adipocytes, which are morphologically indistinguishable from MSCs but have lost their potential to differentiate into other mesenchymal lineages. The second phase is terminal differentiation, in which the pre-adipocytes take on the characteristics of mature adipocytes, i.e., acquiring the machinery for lipid transport and synthesis, insulin sensitivity, and the secretion of adipokines^29^. DEX and GA in normal α-MEM culture medium stimulated the expression of the pre-adipocyte markers *Zfp423* and *Pref-1* in mBMSCs (Fig. 4D), implying that GA had a crucial role in adipocyte commitment. To confirm this, we treated mBMSCs with DEX plus GA (50 μM) or with DEX plus DMSO for 2 d and then continued the culture using full adipogenic medium in the absence of GA or DMSO (treatment scheme shown in Fig. 5A). On day 8, we observed a dramatically higher number of mature adipocytes in the DEX-plus-GA-pretreated group (Fig. 5B), indicating such pretreatment did effectively potentiate adipocyte commitment.

### GA promoted adipocyte commitment, likely through its anti-sumoylation function

Apart from its anti-sumoylation activity, GA has a histone acetylation transferase (HAT) inhibiting activity that inhibits P300/CBP-associated factor (PCAF**)**-mediated histone acetylation *in vitro*^21^. To clarify whether GA modulates mBMSCs adipogenesis through anti-sumoylation or anti-acetylation, we selected for comparison anacardic acid (AA), a sumoylation/HAT inhibitor structurally similar to GA^21^, and garcinol (Gcl), another potent HAT inhibitor reported to block PCAF function^30^. From a 7-d co-treatment with each individual compound plus DEX, we found that both AA and GA promoted, while Gcl inhibited, the adipocyte commitment of mBMSCs (Fig. 5C). Thus, GA was likely regulating adipocyte commitment through its sumoylation-inhibiting activity.

### GA inhibited terminal adipocyte maturation and lipogenesis of mBMSC

**Figure 6:**
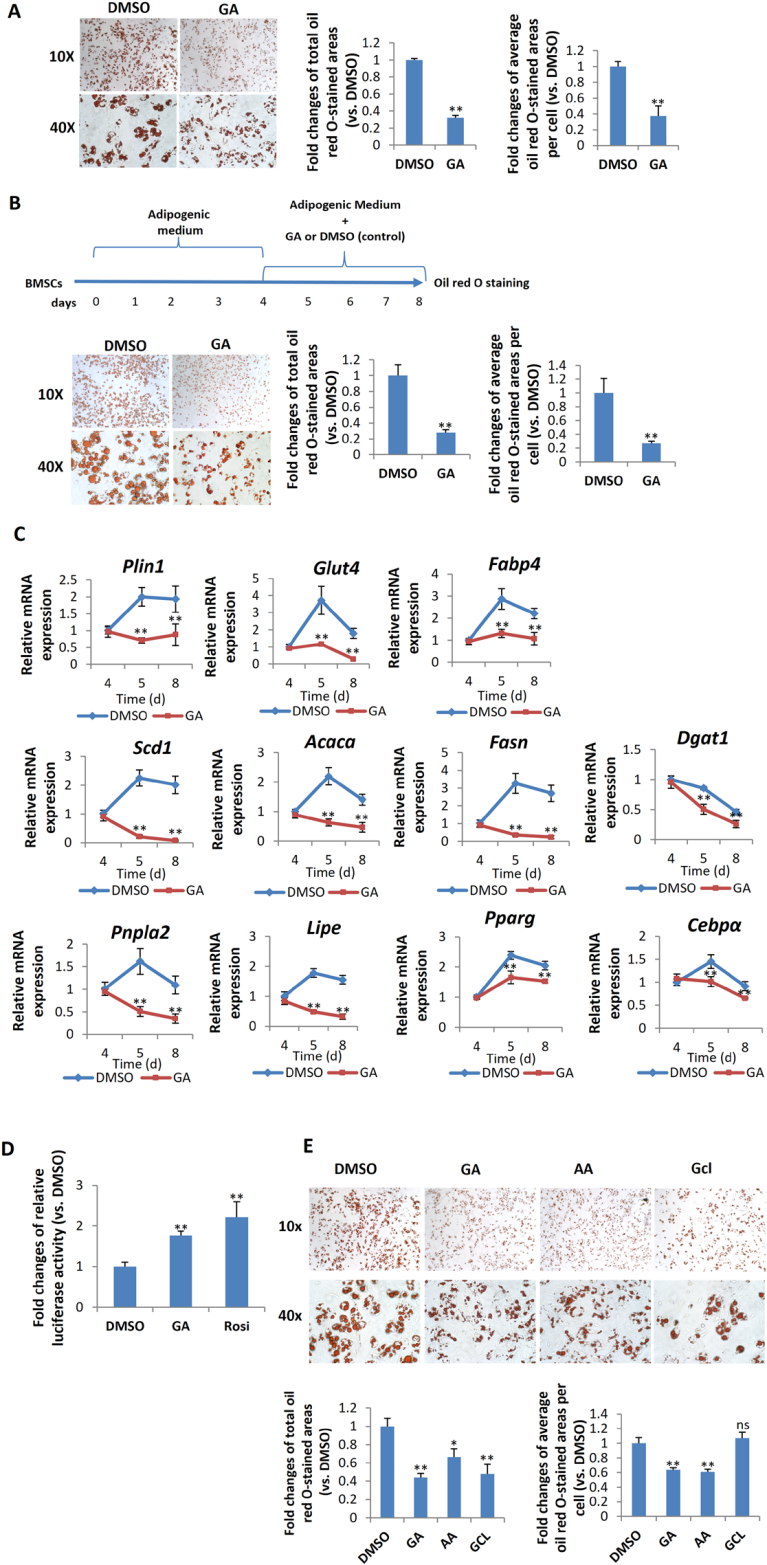
GA inhibited adipocyte maturation of mBMSC. (**A)** mBMSC adipogenic culture treated with GA (50 μM) over the entire differentiation period (7 d). The total oil red O-stained areas and the average oil red O-stained area per cell are quantified on the right (**p < 0.01; n = 4). (**B**) mBMSC adipogenic cultures treated with GA (50 μM) at a later stage (see time line at the top of panel). The cells had dramatically reduced lipid accumulation. The oil red O–stained areas and average stained area per cell are quantified on the right (**p < 0.01; n = 3). (**C**) GA (50 μM) treatments decreased the markers for lipogenesis, lipolysis, mature adipocytes, and master regulators of adipogenesis (**p < 0.01; n = 4). (**D**) PPRE Luciferase reporter assay show increased PPAR activity in GA (50 µM) treated HEK293 cells. Firefly-luminescence intensity was normalized to the Renilla luminescence. Rosiglitason (1 μM) were used as a positive control (**p < 0.01; n = 4). (**E**) As indicated by oil red O staining, mBMSCs cultured in adipogenic medium with GA (50 µM) or AA (50 µM) over the entire differential period (7 d) showed a decrease in lipid accumulation. Gcl (7 µM) treated cells showed decreased total lipid content while the average lipid content in each adipocyte was unchanged. The oil red O–stained areas and average stained area per cell are quantified below (*p < 0.05, **p < 0.01; n = 3).

To evaluate the gross effect of GA on adipocyte differentiation, we treated mBMSCs with GA throughout the process of adipogenesis (for 7 d). GA treatment dramatically decreased the lipid content in differentiated cells (Fig. 6A), implying that GA may inhibit adipocyte maturation. To study GA effects in the later stage of adipogenesis, we treated mBMSCs with normal adipogenic medium for 4 d to initiate early adipogenesis, then continued the culture in the adipogenic medium with GA or DMSO (control) added for another 4 d (treatment scheme shown in Fig. 6B). In comparison with the controls, we found significantly decreased lipid storage in the GA-treated group (Fig. 6B), confirming that GA inhibited terminal adipocyte differentiation by limiting lipid accumulation.

The decreased lipid storage in adipocytes may be a result of decreased lipogenesis, augmented lipolysis, or both^31,32^. Hence, we assessed the expression of lipogenesis- and lipolysis-related genes in the GA-treated adipogenic cultures (treatment scheme is shown in Fig. 6B; cells were harvested for RNA at day 4, 5, and 8). Consistent with the cellular phenotype, we found that the expression of *Plin1* (a lipid droplet coating protein) and of *Glut4* and *Fabp4* (mature adipocyte markers) were dramatically decreased in the GA-treated group at day 5 and day 8 (Fig. 6C). Moreover, key lipogenesis markers including *Scd1*, *Acaca*, *Fasn*, and *Dgat1* were significantly decreased, while lipolysis markers such as *Pnpla2* and *Lipe* were also reduced. The expression of *Pparg* and *Cebpa* were slightly decreased correspondingly (Fig. 6C). These data suggest that GA affected lipid storage primarily by limiting lipogenesis, not by augmenting lipolysis.

It was reported that HATs, such as SRC/p160 and p300/CBP, can activate PPARγ signaling^33–35^, and that PPARγ can be deacetylated and inactivated by deacetylase SIRT1^36,37^. Hence, GA may restrict PPARγ acetylation and activation via its HAT inhibitor activity. However, by a reporter assay (3xPPRE-Luciferase), we found that GA treatment significantly increased the activity of PPARγ (Fig. 6D), suggesting that GA unlikely influences PPARγ signaling via its HAT-inhibitor function. To further determine whether GA’s inhibitory effects on adipocyte maturation were through its anti-sumoylation or anti-HAT activity, AA and Gcl were included in this experiment. Relative to mBMSCs treated with normal adipogenic medium, the GA- or AA-treated cultures decreased the total lipid content (oil-red-positive areas) in the whole well as well as the average lipid content of each adipocyte. However, the Gcl-treated cultures showed lower number of adipocytes but no distinctive change in the average lipid content of each adipocyte (Fig. 6E).

This suggests that GA is similar to AA but distinct from Gcl in regulating lipid storage in the adipocytes, i.e., the lipid storage inhibitory activity of GA and AA likely depends on their anti-sumoylation function. The role of Gcl in the adipogenesis has been tested in the 3T3L1 cells^38^. It is consistent with our findings that Gcl blocks adipocyte commitment and reduced total lipid content in the cultures.

### GA had a similar biphasic function in the adipogenesis of chondrocytes

**Figure 7:**
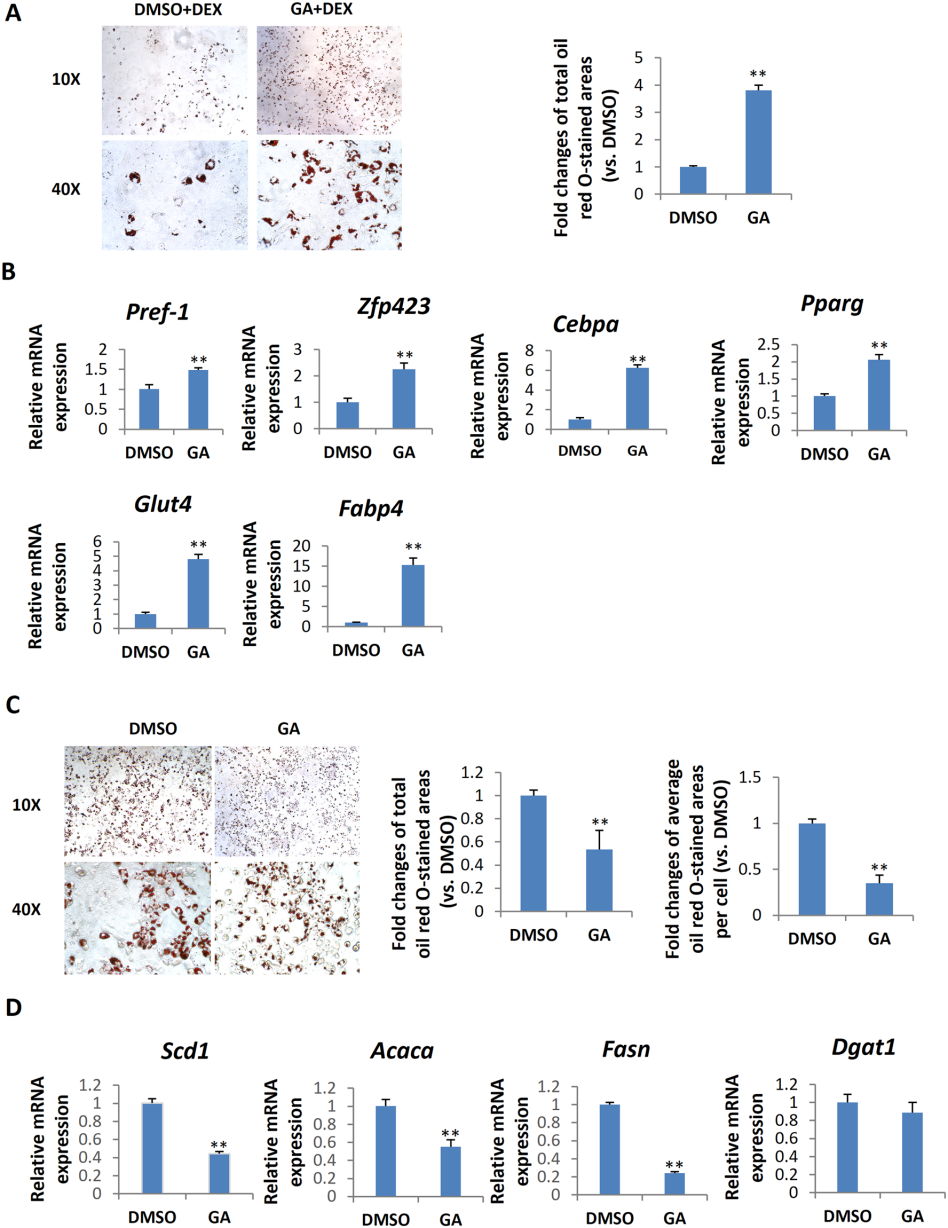
Biphasic effects of GA on chondrocyte adipogenesis. (**A**) Primary rib chondrocyte cultured with normal medium containing Dex plus GA (50 μM) for 7 d. The oil red O–stained areas are quantified on the right (**p < 0.01; n = 3). (**B**) GA (50 μM) treatments increased the markers of adipogenesis (**p < 0.01; n = 3). (**C**) primary rib chondrocyte cultured in the adipogenic medium containing GA (50 μM) over the entire differentiation period (7 d). The oil red O–stained areas and average stained area per cell are quantified on the right (**p < 0.01; n = 3). (**D**) Primary rib chondrocyte cultured in adipogenesis medium for 5 days with GA added at d 5 for 24 h showed a decrease expression of lipogenesis markers (**p < 0.01; n = 4).

Next, we chose chondrocyte to test whether the biphasic effect of GA on adipogenesis is specific to BMSCs or is a relatively common phenomenon. Chondrocytes are known to be able to transdifferentiate into osteoblast and adipocyte under suitable conditions^39–41^. We found that very similar to mBMSCs, the cultured mouse primary rib chondrocytes treated with GA plus DEX had more robust adipogenesis than those treated with DMSO (Fig. 7A). In addition, the expression of pre-adipocyte marker, *Pref-1* and *Zfp423*, were significantly elevated in the chondrocytes treated with GA plus DEX for 3 d (Fig. 7B). Moreover, the mouse primary chondrocytes cultured in the full adipogenic medium containing GA showed a dramatic decrease in the lipid storage (Fig. 7C) and lipogenesis markers (Fig. 7D). These data overall suggest that the biphasic effect of GA in adipogenesis also exists in other cell types besides of mBMSCs.

## Discussion

We have assessed the effect on mesenchymal differentiation of ginkgolic acid, a natural compound extracted from *Ginkgo biloba* leaves that inhibits sumoylation and histone acetyltransferase. We unexpectedly found that during mBMSC osteogenic induction, the addition of GA not only blocked osteoblast differentiation but also dramatically promoted adipocyte formation. These results suggested that GA could steer the fate of MSCs from osteogenesis to adipogenesis.

The GA inhibition of osteoblast formation likely starts at an early stage, because early osteogenic markers such as *Runx2* and *Osx* were decreased. This inhibition is reminiscent of two previous findings regarding the role of sumoylation in osteogenesis: first, PIAS2 knockdown markedly suppresses osteoblast differentiation by inhibiting *Osx* expression^10^; and second, *Ubc9* silencing reduces *Runx2* expression by inhibiting SMAD4- and SMAD1-mediated BMP signaling^11^. However, UBC9 overexpression was also found to inhibit osteoblast differentiation by suppressing BMP/SMAD4 signaling^8^. Further exploration of GA specific targets in osteogenesis is needed.

DEX-plus-GA-treated mBMSCs had increased expression of adipocyte commitment -promoting genes (*Zfp423*, *Pref-1*) and were highly potentiated to adipogenesis. In addition, GA likely acted as a preadipocyte-inducing reagent through its inhibition of sumoylation, not its inhibition of HAT function, because the HAT inhibitor Gcl showed the opposite effect during adipocyte commitment. However, we cannot completely exclude the contribution of GA as a HAT inhibitor in this process, because GA and Gcl may target different HATs. It is also possible that other unidentified functions of GA may contribute to adipocyte commitment. Nevertheless, our finding that GA was a potent pre-adipocyte inducer provides a new experimental reagent for studying adipocyte commitment, which is a much less understood process of adipocyte differentiation.

We also found that GA impaired the later stage of mBMSC adipogenesis by reducing lipogenesis. This reminded us that GA also inhibited the lipogenesis pathway in pancreatic cancer cells and blocked cell growth, suggesting that the role of GA in lipogenesis is conserved in diverse cellular contexts^24^. GA is likely to achieve this through its sumoylation-inhibiting function, because Gcl did not show such an effect in the adipogenesis of mBMSCs. The biphasic effect of GA may partly reflect the multifaceted roles of sumoylation pathway in adipogenesis. Further exploring specific GA downstream regulators that contribute to the two phases of adipogenesis may provide new insights about modulation of adipogenesis by the sumoylation pathway.

Sumoylation regulates a broad spectrum of important cellular processes and diseases and is conceivably a target for disease treatments or tissue regeneration. However, compared with the ubiquitination-modulating compounds that have been widely used clinically^42,43^, sumoylation-modulating compounds have not been extensively explored for medical applications. Recently, GA has been shown to suppress the invasion or growth of lung, breast, and pancreatic cancer cells and has been proposed as a promising anti-cancer drug^22–24^. Here, our work has revealed an unprecedented function of GA in osteogenesis, adipocyte commitment, and lipid accumulation during MSC differentiation. This provides new mechanistic insights of GA in its future clinical applications for lipid-metabolism-related conditions, and raises a concern regarding to its possible side effects in MSC fate determination when used systemically for treatments of cancers or other diseases.

## Materials and Methods

### Mice

C57BL/6 J mice were obtained from Jackson Lab. All mice were maintained and used in accordance with the relevant guidelines and regulations approved by the Van Andel Research Institute Animal Care and Use Committee.

### Chemicals

Ginkgolic acid (15:1) (Calbiochem, Cat# 345887) was dissolved in DMSO to make 50 mM stock (1000×), 50 μM was used for treatment study; Garcinol (Cayman, Cat#, 78824–30–3) was dissolved in DMSO to make 21 mM stock (3000×), 7 μM was used for treatment study; Anacardic acid was purchased from Santa Cruz Biotechnology, Cat# 16611–84–0 and dissolved in DMSO to make 50 mM stock (1000×), 50 μM was used for treatment study. These reagents were diluted with medium to the working concentrations.

### mBMSC and primary mouse rib chondrocyte culture

C57BL/6 J mice (8 weeks) were euthanized by CO_2_. Total bone marrow cells were collected from tibia and femur and then cultured in α-MEM with 10% fetal bovine serum (FBS) in an incubator (37 °C, 5% CO_2_). After 48 h, the non-adherent cells were removed by three washes with PBS, and the adherent cells were continued in culture and used as a source of mBMSCs. The culture medium was replaced every 2 d^44^.

Rib cages from P0 - P3 newborn C57BL/6 J mice were dissected and the soft and bony tissues surrounding the cartilaginous parts of the ribs were removed as much as possible. After washed with PBS for 3 times, the rib cages were incubated in 1 ml of 1 mg/ml pronase (Roche) for 30 min at 37 °C. After washed by PBS for 3 times, ribs were further digested by 1 ml of 1 mg/ml Collagenase Type II (Gibco) in DMEM without serum at 37 °C for 1 h, washed with PBS for 3 times, then incubated in 1 ml of Collagenase Type II (1 mg/ml in DMEM with 10% FBS) overnight in the incubator at 37 °C with 5% CO_2_. The next day, chondrocyte released from the ribs were suspend by gently rocking the dishes, and transferred to tubes, briefly centrifuged and washed with PBS twice, then resuspended and cultured in DMEM with 10% FBS. Medium was replaced every 2 d.

### Osteogenic and adipogenic differentiation

For osteogenic differentiation, mBMSCs were seeded into 24-well plates at 1 × 10^5^ cells/well and cultured in α-MEM. After 80% confluence, the mBMSCs were cultured in osteogenic medium (α-MEM containing 10% FBS, 100 nM dexamethasone, 10 mM β-glycerophosphate, and 0.05 mM L-ascorbic acid-2-phosphate). The osteogenic culture medium was replaced every 2 d. Osteogenic differentiation was evaluated by alkaline phosphatase (ALP) staining and Alizarin Red S staining. For ALP staining, 4 d after differentiation, cells were washed once with PBS and then fixed in 10% formalin for 5 min at room temperature. After three PBS washes, the ALP staining mixture (0.1 mg/ml naphthol AS-MS phosphate, 0.5% *N*,*N*-dimethyl formamide, 2 mM MgCl_2_, 0.6 mg/ml fast blue BB salt, and 0.1 M Tris-HCl [pH 8.5]) was added to the fixed cells for 20 min at room temperature; staining was then stopped by three PBS washes. For Alizarin Red S staining, cell cultures were rinsed once with PBS, fixed in 10% formalin, washed once with tap water, and stained with 2% Alizarin Red S (pH 4.2) for 20 min^44^.

For adipogenic cultures, mBMSCs or chondrocyte were seeded into 24-well plates at 1 × 10^5^ cells/well and cultured in α-MEM. When the cells reached 100% confluence, they were incubated in the adipogenic medium (α-MEM containing 10% FBS, 0.5 mM isobutyl-methylxanthine, 1 μM dexamethasone, 10 μg/ml insulin, and 1 μM rosiglitazone). The culture medium was replaced every 2 d. Adipogenic differentiation was visualized by oil red O staining. Briefly, cells were fixed with 10% formalin for 1 h, rinsed once with 60% isopropanol, and stained for 10 min with oil red O working solution (6 parts of 1% oil red O in 100% isopropyl alcohol mixed with 4 parts of double-distilled water; let sit at room temperature for 20 min and then filter through a 0.22-μm filter). The stained cultures were imaged and total oil red O positive areas and oil red O positive area per oil red O positive cells were calculated using Image J.

### Western blot

Total protein was extracted from HEK293 cells or mBMSCs treated with 50 μM GA for the indicated time using RIPA buffer. Equal amounts of protein lysate from each sample were separated by SDS-PAGE gel and subjected to standard western blot procedures. SUMO2/3 antibody (Sigma, 1:1000) and α-tubulin antibody (Sigma, 1:10000) were used.

### Quantitative RT-PCR

Total RNA was extracted from cell cultures with TRIzol reagent (Invitrogen), purified with an RNeasy kit (Qiagen) according to the manufacturer’s instructions, and used for first-strand cDNA synthesis using a VILO cDNA synthesis kit (Invitrogen). Then the cDNA was analyzed by real-time PCR using SYBR Green QPCR Master Mix (Invitrogen) on a StepOne PCR instrument (Bio-Rad). The relative expression of genes of interest was calculated using the 2^−ΔΔCT^ method; the *Actb* gene was used as an internal control^45^. Primer sequences for qRT-PCR are listed in Table 1.

**Table 1:** PCR primer pairs for qRT-PCR.

Gene	Forward	Reverse
*Runx2*	ATCCCCATCCATCCACTCCA	GCCAGAGGCAGAAGTCAGAG
*Osx*	GTCGGGGAAGAAGAAGCCAA	TAGCAGGTTGCTCTGCTCTG
*Col1a1*	GCACGTCTGGTTTGGAGAGA	ACATTAGGCGCAGGAAGGTC
*Dmp1*	CTTGTGTTCCTTTGGGGGCT	GACTCACTGTTCGTGGGTGG
*Sost*	CCTCCCCACCATCCCTATGA	GTCAGGAAGCGGGTGTAGTG
*Pparg*	TTGCTGTGGGGATGTCTCAC	AACAGCTTCTCCTTCTCGGC
*Cebpa*	TCGGTGGACAAGAACAGCAA	TGGTCAACTCCAGCACCTTC
*Fabp4*	GGTGCAGAAGTGGGATGGAA	CTCTTGTGGAAGTCACGCCT
*Glut4*	AGCGAGTGACTGGAACACTG	TCAATCACCTTCTGTGGGGC
*Plin1*	CCCGGCTCTTCAATACCCTC	ATGGGCACACTGATGCTGTT
*Pref1*	CCTGGCTGTGTCAATGGAGT	CAAGTTCCATTGTTGGCGCA
*Zfp423*	CGCGATCGGTGAAAGTTGAA	CGATCACACTCTGGCTCTCC
*Scd1*	TGGAGTACGTCTGGAGGAACA	GCGCTGGTCATGTAGTAGAAAATC
*Acaca*	ACGTGCAATCCGATTTGTTGT	TGTTGTTGTTGGGTCCTCCA
*Dgat1*	CCGGGACAAAGACGGGC	ACCACGATAATTGCTGAAACCAC
*Fasn*	AAGCAGGCACACACAATGGA	CAGTGTTCGTTCCTCGGAGT
*Lipe*	AAAAGACCACATCGCCCACA	CTGCCTCAGACACACTCCTG
*Pnpla2*	AACGCCACTCACATCTACGG	AATGTTGGCACCTGCTTCAC
*Actb*	TTTGCAGCTCCTTCGTTGC	ACGATGGAGGGGAATACAGC

### RNA sequencing and data analysis

mBMSCs were cultured in normal medium with GA or DMSO (control) plus DEX for 3 d. Total RNA was extracted using the methods described above and sequenced using the Novogene RNA-seq service (Hongkong, China). RNA-seq results were further analyzed through Gene Set Enrichment Analysis (GSEA) and DAVID (http://david.abcc.ncifcrf.gov/). Heat maps were generated and hierarchically clustered using MeV software ver. 4.9. Genes selected for comparison in the heat map were differentially expressed between GA and DMSO treatment (genes had a log_2_ fold change >0.5 or <–0.5 were chosen for analysis; p < 0.05).

### Reporter assay

HEK293 cells (ATCC) at 60–70% confluency cultured in 24-well plates were transfected with 500 ng 3xPPRE-luciferase plasmid (Addgene) and 50 ng pRL-Renilla plasmid (Addgene, for normalization of transfection efficiency) using the X-fect transfection reagent (Clontech). After 24 h of culture, these cells were treated with 50 μM GA, DMSO, or 1 μM rosiglitazone (positive control) (n = 3). The cell lysate was collected for luciferase assay using a Dual-Luciferase kit (Promega) and measured on a Biotek Synergy plate reader. The firefly-luminescence results were normalized to the corresponding Renilla luminescence.

### Statistical Analysis

All statistical results were achieved by applying two-tailed, unpaired Student’s *t*-test; a P value lower than 0.05 was considered statistically significant. The data were presented as mean values +/– standard deviation (S.D.). Each cell differentiation or qRT-PCR study that used primary mBMSCs and chondrocytes had at least 3 biological samples (n ≥ 3; the primary cells from individual mouse were considered one biological sample); 2–3 technical repeats were performed for each biological sample and the mean value represented the result of that sample. For Western blots, mBMSCs from 3 mice were cultured separately (n = 3), treated with GA, harvested at designated time points. The Western blots results were quantified for statistical analysis.

### Data availability

RNAseq data that support the findings of this study have been deposited in Gene Expression Omnibus with the accession code GSE104911 (https://www.ncbi.nlm.nih.gov/geo/query/acc.cgi?acc=GSE104911).
